# Glymphatic System Dysfunction in Central Nervous System Diseases and Mood Disorders

**DOI:** 10.3389/fnagi.2022.873697

**Published:** 2022-04-25

**Authors:** Dianjun Zhang, Xinyu Li, Baoman Li

**Affiliations:** ^1^Department of Forensic Analytical Toxicology, School of Forensic Medicine, China Medical University, Shenyang, China; ^2^Liaoning Province Key Laboratory of Forensic Bio-evidence Sciences, School of Forensic Medicine, China Medical University, Shenyang, China; ^3^China Medical University Center of Forensic Investigation, School of Forensic Medicine, China Medical University, Shenyang, China

**Keywords:** glymphatic system, mood disorder, astrocyte, sleep, central nervous system disease

## Abstract

The glymphatic system, a recently discovered macroscopic waste removal system in the brain, has many unknown aspects, especially its driving forces and relationship with sleep, and thus further explorations of the relationship between the glymphatic system and a variety of possible related diseases are urgently needed. Here, we focus on the progress in current research on the role of the glymphatic system in several common central nervous system diseases and mood disorders, discuss the structural and functional abnormalities of the glymphatic system which may occur before or during the pathophysiological progress and the possible underlying mechanisms. We emphasize the relationship between sleep and the glymphatic system under pathological conditions and summarize the common imaging techniques for the glymphatic system currently available. The perfection of the glymphatic system hypothesis and the exploration of the effects of aging and endocrine factors on the central and peripheral regulatory pathways through the glymphatic system still require exploration in the future.

## Introduction

The “glymphatic system” (glial-lymphatic system), which was proposed by Jeffrey Iliff, Maiken Nedergaard and her team in 2012, mainly refers to a macroscopic waste clearance system similar to the peripheral lymphoid system in the brain (Iliff et al., 2012). Numerous studies have shown that the glymphatic system exerts a significant effect on the distribution of a variety of substances in the brain, including not only its core waste clearance function but also the distribution and transport of nutrients such as glucose (Lundgaard et al., 2015), some possible treatment substances (recombinant adeno-associated viral vectors) (Murlidharan et al., 2016) and glial calcium signaling (Rangroo Thrane et al., 2013). Because of its important physiological role, the glymphatic system is considered related to the occurrence and development of a variety of diseases (Rasmussen et al., 2018), including neurodegenerative diseases such as Alzheimer’s disease, Parkinson’s disease, as well as mood disorders (Yan et al., 2021).

The brain contains a very rich and complex network of blood vessels. Most of the blood vessels in the brain parenchyma are wrapped by the protruding processes (endfeet) of astrocytes (Nagelhus and Ottersen, 2013; Korogod et al., 2015). The perivascular space (PVS) between the astrocytic endfeet and the vascular wall is filled with cerebrospinal fluid (CSF), which is the main site of action of the glymphatic system. The water channel aquaporin-4 (AQP4) occupies approximately 50% of the surface area of astrocyte processes located close to or in direct contact with blood vessels (Nielsen et al., 1997). CSF passing through the subarachnoid space enters the stroma through AQP4 located in the space around the artery, pushes the interstitial fluid (ISF) to carry solute to the perivenous space, and then flows out of the brain into the cervical lymphatic system (Murtha et al., 2014). Relying on the PVS, astrocytes are the core, and the AQP4-dependent material transport system is the glymphatic system.

The main factors influencing the glymphatic system include AQP4, sleep and arterial pulsation (Jessen et al., 2015). However, the role of arterial pulsation in it is still open to question. AQP4 is a small hydrophobic intrinsic plasma membrane protein, and its physiological function is to promote the transport of water through the plasma membrane. Because its expression on astrocytes is highly polarized (mainly concentrated on the capillary surface of the endfeet) (Nielsen et al., 1997), it reduces the resistance of CSF transport between the PVS and interstitial space (Iliff et al., 2012). Therefore, astrocytes have the ability to guide the transformation of CSF and ISF into each other in both the space around the arteries and veins.

Some studies have shown that the glymphatic system is inhibited in the awake state but enhanced in the sleep state (Xie et al., 2013). Sleep may affect the glymphatic system by changing the volume of the brain stroma. The brain interstitial volume fraction in asleep and anesthetized mice was higher than that in the awake state, which reduced the resistance of CSF flowing into the brain stroma and was beneficial to the operation of the glymphatic system (Xie et al., 2013). In addition, norepinephrine is a major neurotransmitter involved in the awakening state (Berridge and Waterhouse, 2003), and a sudden increase in its levels during awakening increases the cell volume in the brain parenchyma and may resist the inflow of CSF by reducing the interstitial volume (O’Donnell et al., 2012). Although controversy persists regarding whether the choroid plexus is the source of CSF production (Oresković and Klarica, 2010; Trillo-Contreras et al., 2019), several studies have confirmed that norepinephrine directly acts on choroid plexus epithelial cells to inhibit CSF production (Nilsson et al., 1992), which may be part of the mechanism of the sleep inhibitory glymphatic system.

Generally, intracranial arterial pulsation is presumed to be the main physical force promoting the operation of the glymphatic system (Jessen et al., 2015). Related experiments have indicated that the efficiency of CSF flowing into the brain is positively correlated with the pulsation of arteries (cortical penetrating arteries) (Iliff et al., 2013b). Every 10-beat reduction in heart rate reduces waste clearance and leads to an approximately 20% increase in β-amyloid protein (Aβ) levels in the brain parenchyma, while an increase in heart rate of 30 beats per minute produced an approximately 30% decrease in Aβ levels in the brain parenchyma (Kyrtsos and Baras, 2015). Although the glymphatic system is most active during sleep (Xie et al., 2013), blood pressure, cerebral blood flow (Kotajima et al., 2005) and vascular movement (Zhang and Khatami, 2014) are all at a low level, which contradicts this conclusion. However, a study by Goulay et al. (2018) in non-human primates confirmed that solute clearance in the brain is severely impaired in the presence of CSF leakage, supporting a link between CSF circulation and glymphatic system. Therefore, the relationship between arterial pulsation and the glymphatic system is not fully understood.

This review summarizes and introduces the research progress on understanding the role of the glymphatic system in central nervous system diseases and mood disorders, discusses the possible mechanisms underlying the occurrence and development of related diseases. We focus on the abnormalities in various components of the glymphatic system and the role of sleep in the development of the disease, combined with summarizing the commonly used visual imaging methods, to provide strategies for the prevention and treatment of related diseases in the future.

## Central Nervous System Diseases

### Neurodegenerative Diseases

Neurodegenerative diseases (NDDs) are diseases characterized by progressive dysfunction and neuronal loss (Kovacs, 2016). Common NDDs include Alzheimer’s disease (AD), Parkinson’s disease (PD), amyotrophic lateral sclerosis (ALS) and Huntington’s disease (HD). A possible cause of these destructive diseases is the abnormal aggregation and deposition of toxic proteins such as Aβ, α-synuclein, TAR-DNA-binding protein 43 and mutant huntingtin protein respectively (Kovacs, 2016; Ruz et al., 2020). This pathological deposition occurs not only in the brain but also in peripheral organs (Kovacs, 2019) and affects neurons and glial cells simultaneously (Kovacs, 2016).

#### Alzheimer’s Disease

Alzheimer’s disease is a relatively slowly progressing neurodegenerative disease. As a common type of dementia, its main pathological features are the deposition of senile plaques and neurofibrillary tangles (NFTs) (De-Paula et al., 2012; Tarasoff-Conway et al., 2015). Senile plaques (also known as neuritic plaques) are attributed to the extracellular deposition of Aβ, and the accumulation of high-density plaques in the hippocampus, amygdala and cerebral cortex leads to the activation of astrocytes and microglia, synaptic loss, and cognitive impairment (Tabaton and Piccini, 2005; Armstrong, 2009; Chen et al., 2017). Aβ deposition also ossifies blood vessels and reduces arterial pulsation, leading to cerebral amyloid angiopathy (CAA) (Serrano-Pozo et al., 2011). Tau is a splice variant of microtubule-associated protein tau (MAPT), which is an intracellular neuronal protein that stabilizes axons (Shea and Beermann, 1994). In patients with AD, intracellular tau (i-tau) is hyperphosphorylated, resulting in the formation of insoluble neurofibrillary tangles that are difficult to remove (Binder et al., 2005).

Schubert et al. (2019) uses MRI and dynamic ^11^C-PiB PET to prove that CSF clearance deficits in AD associated with Aβ deposits. A study using ultrafast 10 Hz magnetic resonance encephalography also showed that pervasive abnormality in (peri) vascular cerebrospinal fluid impulse propagation exists in patients with AD, which can damage glymphatic brain clearance of Aβ in AD (Rajna et al., 2021). The glymphatic system is inhibited in an AD (APP/PS1) mouse model, and this inhibition occurs with or without showing extensive Aβ deposits, due to the accumulation of toxic Aβ species, such as soluble oligomers (Peng et al., 2016), this indicates that there may be a complex interaction relationship between AD and glymphatic system. Using transgenic mice, Patel et al. (2019) confirmed that the glymphatic system plays an important role in the transport and clearance of extracellular tau protein. As the functional core of the glymphatic system, AQP4 promotes the clearance of the interstitial aggregation of proteins, including Aβ and tau (Mader and Brimberg, 2019). Two-photon imaging studies showed that the Aβ clearance rate of AQP4 knockout mice is decreased by 55–65%(Iliff et al., 2012), indicating that AQP4 plays an important role in the clearance of extracellular Aβ (EAβ) in mice. The pharmacological inhibition of AQP4 (AQP4 inhibitor TGN-020) also significantly reduces the tau clearance rate (Harrison et al., 2020). Using human transcriptomic data, researchers demonstrated that the expression of perivascular astroglial gene products are associated with phosphorylated tau levels in temporal cortex (Simon et al., 2018). However, under the pathological condition of AD, AQP4 may be abnormal. The lower concentrations of Aβ(1-42) (a soluble monomeric isomer of Aβ; Zheng et al., 2012) increased AQP4 expression in cultured mouse cortical astrocytes, while the higher concentrations of Aβ(1-42) decreased AQP4 expression (Yang et al., 2012). AQP4 depolarization was observed in the brains of patients with AD (Zeppenfeld et al., 2017) and AD model mice (Xu et al., 2015). These deletions or changes in the location of polarization substantially inhibit the clearance function of the glymphatic system, resulting in the accumulation of large amounts of Aβ and tau in the brain, leading to the continuous deterioration of AD.

One of the characteristics of patients with AD is sleep disturbance (Moran et al., 2005). Sleep-wake cycle disorders caused by Aβ accumulation and loss of diurnal fluctuations in Aβ levels in ISF are observed in mice and humans (Roh et al., 2012), while the glymphatic system is most active during sleep. The Aβ clearance rate in the brain of sleeping mice is twice as fast as that of awake mice (Xie et al., 2013), and Aβ deposition in the human brain increases significantly after sleep deprivation for one night (Shokri-Kojori et al., 2018). Based on these results, sleep plays an important role in Aβ clearance. Another study showed that people with poor sleep habits and specific genetic variations in AQP4 have more Aβ deposits in the brain (Rainey-Smith et al., 2018), suggesting that AD may inhibit glymphatic system function through both AQP4 abnormalities and sleep disorders, resulting in protein accumulation and related pathological changes.

#### Parkinson’s Disease

Parkinson’s disease is a common neurodegenerative disease, and its symptoms mainly include motor disorders (neuromuscular stiffness, motor retardation, and tremor) and non-motor disorders (sleep disorders, constipation, and malnutrition) (Henderson et al., 2019). The main pathological feature of PD is the loss of dopaminergic neurons in the substantia nigra, accompanied by the accumulation of α-synuclein (α-syn) in Lewy bodies (Bohnen and Hu, 2019). Blocking meningeal lymphatic drainage by ligating the deep cervical lymph nodes in mice has also been shown to aggravate the pathological and motor symptoms caused by α-syn (Zou et al., 2019), indicating a possible relationship between the glymphatic system and PD.

α-Syn is considered a protein that regulates synaptic neurotransmitter release and autophagy (Bobela et al., 2015), and its deposition in the brain is the basis of the neuropathology of PD. α-Syn accumulates in many parts of the brain, causing neuroinflammation. The interaction between neuroinflammation and systemic oxidative stress may exacerbate reactive astrogliosis and potential damage to the glymphatic system (Verkhratsky et al., 2015; Chiang et al., 2018), while the neurotoxicity of α-syn itself promotes the loss of dopaminergic neurons in the substantia nigra-striatum circuit, leading to related symptoms (Taylor et al., 2002; Kovacs et al., 2008). Some experiments have shown a negative correlation between α-syn deposition and AQP4 expression (Hoshi et al., 2017), while a decrease in AQP4 expression aggravates the degeneration of dopaminergic neurons (Zhang et al., 2016). To some extent, this finding explains the possible pathological relationship between the glymphatic system and PD.

Sleep disorders and poor sleep quality are strongly associated with decreased cognitive performance in patients with PD (Stavitsky et al., 2012; Bohnen and Hu, 2019). One of the common symptoms is rapid eye movement-sleep behavior disorder (RBD), a sleep disorder characterized by a lack of muscle tone, which leads to abnormal body movement during REM sleep and often occurs before motor symptoms (Barber and Dashtipour, 2012). A study of autopsy samples showed a cumulative increase in α-syn levels in many regions of the brains of patients with PD presenting sleep disorders (Kalaitzakis et al., 2013), and evidence that sleep dysfunction in patients with PD is caused by the degeneration of dopaminergic neurons in a pathway similar to RBD has been reported (Dickson et al., 2009; Kalaitzakis and Pearce, 2009; Grinberg et al., 2010). Dopamine, similar to norepinephrine, increases alertness and promotes arousal, and its levels increase mainly in the state of awakening (Isaac and Berridge, 2003; Murillo-Rodríguez et al., 2009). There is a possible interaction between dopamine and clock gene expression, and dopaminergic dysfunction may lead to circadian rhythm disorder (Imbesi et al., 2009; Albrecht, 2013). In addition, some experiments have shown that dopamine reduces the expression of AQP4 in striatal astrocytes, and this downregulation causes the dopamine-induced decrease in proliferation of these cells (Küppers et al., 2008). Based on a large number of experimental results, researchers speculated a vicious cycle, which is the combined neurotoxic effects of α-syn and dopaminergic neurons lead to glymphatic system dysfunction by causing REM sleep abnormalities, circadian rhythm and clock gene dysfunction, while the decrease of α-syn clearance furthers the PD degenerative process (Sundaram et al., 2019).

#### Amyotrophic Lateral Sclerosis

Amyotrophic lateral sclerosis (ALS) is a rapidly progressive neurodegenerative disease characterized by the death of supraspinal and supraspinal neurons responsible for controlling various muscles (Kiernan et al., 2011). Many proteins are related to ALS, such as superoxide dismutase 1 (SOD1), fusion sarcoma (FUS) and Aaxin-2, among which TAR-DNA-binding protein 43 (TDP-43) is the most relevant; it binds RNA and regulates mRNAs involved in neuronal development (Geuens et al., 2016; Prasad et al., 2019). TDP-43 inclusion bodies are widely regarded as pathological markers of ALS, and more than 95% of patients with ALS show TDP-43 deposition (Neumann et al., 2006). TDP-43 deposition represents a possible glymphatic disorder, and the observation of AQP4 overexpression (Nicaise et al., 2009; Dai et al., 2017) and depolarization (Dai et al., 2017) in mouse or rat ALS models supports this speculation. AQP4 depolarization is consistent with the progression of ALS, and its persistence will interfere with neuronal function (Dai et al., 2017). A recent study observed that the cervical spinal cord clearance of the fluorescent dye-labeled tracer (ovalbumin) injected through the cisterna magna of ALS model SOD1^G93A^ mice decreased by approximately 35% compared with wild-type mice, suggesting that ALS model mice have a dysfunctional glymphatic system (Hirose et al., 2021). Meng et al. (2019) temporarily increase the permeability of the blood-brain barrier, recording gadobutrol patterns in the PVS, subarachnoid space, and space surrounding large cortical draining veins in ALS patients, suggesting glymphatic efflux persists in humans. Another pathological feature of ALS is reactive glial hyperplasia characterized by astrocyte hypertrophy and microglial proliferation (Ng et al., 2015), which may also indicate potential glymphatic system damage.

In addition, sleep quality and respiratory function are negatively correlated with the severity of ALS (Ahmed et al., 2016). Increased norepinephrine concentrations have been detected in the CSF, plasma and spinal cord tissues (thoracic and lumbar spinal cord) of patients with ALS (Bertel et al., 1991), which may also affect the glymphatic system.

### Traumatic Brain Injury

Traumatic brain injury (TBI) increases the risk of other neurological and mental disorders, including chronic traumatic encephalopathy, AD, anxiety, depression and personality disorders (Bolte and Lukens, 2021). TBI induces the release of Aβ and C-tau. C-tau is a biomarker of brain injury, and its release is related to the severity of TBI (Gabbita et al., 2005). Several methods (AQP4 gene knockout, cisterna magna cisternostomy, acetazolamide treatment, and sleep deprivation) have been used to inhibit the function of the glymphatic system in the mouse brain. The plasma levels of three biomarkers of TBI [S100β, glial fibrillary acidic protein (GFAP) and neuron-specific enolase] are decreased, indicating that these biomarkers leave the brain through the glymphatic system (Plog et al., 2015), while this glymphatic system-dependent process slows down after TBI (Iliff et al., 2014). In patients with TBI, astroglial inflammation, C-tau, p-tau and Aβ protein waste accumulation and other pathological manifestations are considered to lead to glymphatic system dysfunction (Jessen et al., 2015), and subarachnoid hemorrhage caused by head trauma is also considered to damage the glymphatic system (Gaberel et al., 2014). Inglese et al. (2005) used MRI to evaluate the structural abnormalities of the brain of 24 patients with mild TBI, compared with healthy control subjects, the number of dilated Virchow-Robin spaces (VRS) in the brain of patients with mild TBI increased, indicating the potential dysfunction of glymphatic system. Experiments have suggested that TBI can lead to the clearance of radioactive tracers from the mouse brain: the function of the glymphatic system is reduced by approximately 60% and lasts for at least 28 days (Iliff et al., 2014). This dysfunction of the glymphatic system is related to the depolarization of AQP4 (Ren et al., 2013; Mestre et al., 2018) and has a certain regional specificity (Christensen et al., 2020). As the severity of TBI increases, the recovery time of the AQP4 polarization distribution is correspondingly prolonged (no recovery is observed after 28 days) (Ren et al., 2013). TBI is also related to the formation of astrocyte scars and the continuous activation of neuroinflammation (Iliff et al., 2014). Inflammation and proliferation of reactive astrocytes and microglia appear around the injured site (Giza and Hovda, 2014), while the polarization of AQP4 on the astrocyte endfeet seems to be closely related to the proliferation of reactive astrocytes.

In addition, sleep disorder is one of the common complaints in patients with head trauma. Somnolence, insomnia, and other symptoms appear in a large number of patients with TBI (Clinchot et al., 1998; Ouellet et al., 2006; Verma et al., 2007; Collen et al., 2012), suggesting that TBI damages the function of the glymphatic system by affecting patients’ normal sleep and subsequently resulting in a large accumulation of related biomarkers in the brain.

### Stroke

Stroke is a group of diseases that cause brain damage due to the sudden rupture of cerebral blood vessels or vascular obstruction, including ischemic stroke and hemorrhagic stroke. In mice, both ischemic stroke and hemorrhagic stroke have been shown to lead to the blockage of CSF circulation and damage to the glymphatic pathway (Iliff et al., 2014). Goulay et al. (2017) reported a similar phenomenon in non-human primates: after subarachnoid hemorrhage (SAH), the deposition of fibrin and fibrinogen in the PVS leads to glymphatic system dysfunction and finally the deterioration of cerebral ischemia and brain edema; the application of tissue-type plasminogen activator to remove the deposits in the PVS alleviates the pathological symptoms of SAH. Liu et al. (2021) used routine MRI, diffusion tensor images scan of the brain and other methods to measure the ALPS-index and other indexes of patients with carotid plaque, which confirmed that there may be impairment of glymphatic system in patients with carotid plaque.

Post-SAH glymphatic system dysfunction significantly reduces the amount of CSF flowing into and out of the brain (Liu E. et al., 2020) and causes pathological manifestations such as vasculitis and neuroinflammation (Luo et al., 2016). The main mechanism may be the destruction of the blood brain barrier after stroke (Yang and Rosenberg, 2011). Aβ infiltrates into the CSF and brain parenchyma (Scheuner et al., 1996) and accumulates transiently in the brain parenchyma and blood vessel walls (Garcia-Alloza et al., 2011; Okamoto et al., 2012), leading to inflammation. A study has shown significantly increased AQP4 expression in the artery after SAH, but no significant change in AQP4 expression was detected in the vein, which may indicate a significant increase in the inflow of CSF from the periarterial space, while the volume of ISF expands due to the constant expression of AQP4 in the vein to eventually lead to the deterioration of brain edema (Liu E. et al., 2020). Researchers have observed increased AQP4 expression during the response to hypoxia: acute hypoxia induces AQP4 localization in cortical astrocytes and increases water permeability (Kitchen et al., 2020). This result confirms the findings reported by Mestre et al. (2020) that the entry of CSF into the brain through the glymphatic pathway is the main mechanism of edema formation and ion disturbance in the early stage of ischemic stroke. Other researchers have found that the function of the glymphatic system changes over time after ischemic stroke, and CSF inflow increases a few minutes after stroke but remains relatively slow in the following period (until 7 days) (Lin et al., 2020). Moreover, local destruction of the glymphatic system was detected around the infarct area, while the extracellular fluid in the infarct area was still toxic to neurons 7 weeks after stroke, causing long-term damage because it was not cleared in a timely manner (Zbesko et al., 2018).

Cerebral apoplexy-induced damage to the function of the glymphatic system may also lead to accelerated deposition of Aβ, tau and other proteins, which in turn trigger neurodegenerative diseases such as AD, dementia, and cognitive impairment.

### Idiopathic Normal Pressure Hydrocephalus

Idiopathic normal pressure hydrocephalus (iNPH) is a neurological disease characterized by Hakeem’s triad (dementia, gait disorder, and urinary incontinence) (Lu et al., 2020). Its typical imaging findings are ventricular enlargement, wide lateral fissure, and subarachnoid stenosis (Capone et al., 2020), but intracranial pressure usually remains normal (Hakim and Adams, 1965). INPH is considered one of the most common causes of dementia and one of the risk factors for the subsequent development of AD (Ringstad et al., 2017). Different studies have used enhanced MRI to observe delayed clearance of the intrathecal CSF tracer gadobutrol in patients with iNPH (Ringstad et al., 2017; Eide and Ringstad, 2019). Yokota et al. (2019) performed diffusion tensor imaging (DTI) along the perivascular space (ALPS) and observed lower diffusivity ALPS in patients with iNPH than in the control group. Other groups reported delayed enrichment and clearance of tracers in the visual pathway of patients with iNPH (Jacobsen et al., 2020).

Glymphatic system dysfunction is a component of the pathological process of iNPH. Many possible mechanisms of glymphatic system dysfunction in iNPH pathology have been proposed. One is that excess CSF will be trapped in the dilated PVS and oppress the penetrating artery in the parenchyma of the brain to reduce its pulsation (Gallina et al., 2020). As a possible driving force of the glymphatic system, the inhibition of arterial pulsation may reduce the function of the glymphatic system. Second, decreased AQP4 expression in astrocytes of patients with iNPH mainly results in a decrease in the density of the endfeet around the blood vessels (depolarization) (Eide and Hansson, 2018; Reeves et al., 2020). As a disease related to aging, the prevalence rate of iNPH increases exponentially with age (Martín-Láez et al., 2016). The progressive depolarization of AQP4 caused by aging will further inhibit the operation of the glymphatic system (Hasan-Olive et al., 2019). Finally, patients with iNPH are often diagnosed with sleep disorders (Román et al., 2019). Obstructive sleep apnea (OSA) is a common sleep disorder associated with AD (Ooms and Ju, 2016), affecting 90.3% of patients with iNPH in one study (Román et al., 2019). Abnormal respiratory movement (intermittent airway obstruction) in people with OSA may increase intracranial venous pressure by increasing intrathoracic negative pressure and reducing venous reflux, perivenous CSF outflow and CSF-ISF exchange (Román et al., 2019), and may damage the glymphatic system while promoting the development of iNPH.

## Sleep and Mental Disorders

According to recent studies, mood disorders are also associated with the glymphatic system (Xia et al., 2017b; Liu X. et al., 2020). Mood disorders are a group of mental disorders characterized by emotional imbalance, including major depressive disorder (MDD) and bipolar disorder (BD). In addition to emotional abnormalities, sleep abnormalities are also strongly associated with symptoms associated with mood disorders (Jackson et al., 2003; Lyall et al., 2018). Sleep disorder is a common complaint of patients with mood disorders, while paradoxical sleep deprivation (PSD) causes manic-like behavior in mice (Arent et al., 2015), induces mania in healthy subjects, and exacerbates manic episodes or lead to the transition from depression to mania in patients with bipolar disorder (Kaplan and Harvey, 2013). In addition, some antidepressant drugs, such as ketamine, exert an effect on sleep patterns (Duncan et al., 2017). The aforementioned evidence proves that sleep disorders play an important role in the occurrence and development of mood disorders, and because of the close relationship between sleep and the glymphatic system, we speculate that glymphatic system dysfunction is one of the possible pathological mechanisms of mood disorders. Due to altered sleep patterns or quality, glymphatic system function is abnormal, which leads to or aggravates mood disorders.

### Sleep Disorder

The relationship between sleep and the glymphatic system is inseparable. During non-rapid eye movement (NREM) sleep, a consistent pattern of slow wave activity and CSF inflow is observed, supporting the exciting possibility that sleep regulates glymphatic function (Fultz et al., 2019). Siow et al. (2021) used diffusion tensor imaging-analysis along the perivascular space (DTI-ALPS) index as the MRI marker of glymphatic function, identified significant associations between glymphatic function and sleep in 84 elderly participants. However, the specific interaction between the glymphatic system and sleep remains unclear. Circadian rhythm is a natural internal process that regulates the sleep/wake cycle and repeats roughly every 24 h. Sleep/wake cycle refers to a physiological state that occurs alternately between sleep and awakening. Although previous experiments have shown that the glymphatic system clearance function is related to animal sleep/awakening states (Xie et al., 2013), recent experiments have shown that the glymphatic system is regulated by circadian rhythm rather than the sleep/wake cycle (Cai et al., 2020; Hablitz et al., 2020), which means that glymphatic system may also be affected by endogenous hormones, not just by sleep/awakening state. The redistribution of intraventricular contrast agent in conscious rats was the lowest in the light stage and the highest in the dark phase in both normal and reverse light-dark cycles, which proved the possible effect of alternating day and night cycles on the clearance function of the glymphatic system (Cai et al., 2020). The clearance function of the glymphatic system peaks at the most likely time mice sleep (noon) during the day, and the diurnal difference in glymphatic system function persists under constant light conditions, suggesting that this diurnal difference arises from endogenous regulation (Hablitz et al., 2020). At the same time, the distribution of polarized AQP4 on the endfeet of astrocytes is also related to different periods of the same day, and deletion of the AQP4 gene effectively eliminates the diurnal regulation of the CSF distribution (Hablitz et al., 2020).

Regardless of whether the glymphatic system is regulated by circadian rhythm, the sleep/wake cycle or both, circadian rhythms and sleep/wake cycles in patients with mood disorders have both been shown to be affected to varying degrees (Kalmbach et al., 2015; Bradley et al., 2017; Park et al., 2018). Symptoms of depression show diurnal patterns that are worse in the morning or at night (Rusting and Larsen, 1998), while genomic studies have further discovered the relationship between circadian rhythms and mood disorders. The circadian rhythm gene CRY2 has been proven to be related to the rapid cycling of BD (Sjöholm et al., 2010). The abnormal expression of circadian rhythm genes such as BMAL1, PER1-3, REV-ERBA, DBP, and BHLHE40/41 was also observed in the postmortem brain tissues of patients with MDD (Li et al., 2013). Sufficient studies on the sleep/wake cycle have proven the relationship between mood disorders and this cycle. As shown in our previous study, fluoxetine, a specific serotonin reuptake inhibitor widely used in the clinic, increases the activity of signal transducer and activator of transcription-3 (STAT3) in astrocytes, reverses the changes in the expression and function of nucleotide-binding domain and leucine-rich repeat protein-3 (NLRP3) inflammasomes stimulated by sleep deprivation, and then inhibits SD-induced neuroinflammation and neuronal apoptosis (Xia et al., 2017a). Sleep disorders in patients with mood disorders are likely to affect the function of the glymphatic system, but the significance of this alteration in the occurrence and development of mood disorders remains unclear.

### Mood Disorders

We observed that AQP4 expression was downregulated in the cortex and hippocampus of a mouse model of chronic unpredictable mild stress (CUMS), the widespread loss distribution of AQP4 on astrocytes suggesting a disorder in the glymphatic system, and an increase in Aβ deposition was detected (Xia et al., 2017b). In addition, this disorder was improved by fluoxetine and polyunsaturated fatty acids (PUFAs) (Xia et al., 2017b; Liu X. et al., 2020). Our previous experimental results have shown that iron dextran damages the glymphatic system in the frontal cortex by increasing reactive astrogliosis, which was indicated by an increase in glial fibrillary acidic protein expression, inducing neuronal apoptosis and therefore aggravates depression-like behavior caused by CUMS (Liang et al., 2020). Decreases in the number of astrocytes and AQP4 expression were observed in other animal models of depression, such as chronic mild stress (CMS) (Gong et al., 2012) and chronic psychosocial stress (Czéh et al., 2006). Reactive astrocyte proliferation and changes in AQP4 expression are common in postmortem tissues from patients with depression, but the related experiments have produced contradictory results. Glial fibrillary acidic protein (GFAP) is one of the specific biomarkers of astrocytes. Several experimental results show that depression reduces its expression in different regions of the brain (Altshuler et al., 2010; Bernard et al., 2011; Gittins and Harrison, 2011; Cobb et al., 2016; Torres-Platas et al., 2016), but some experiments show that its expression increases (Barley et al., 2009) or it not significantly changed (Williams et al., 2013; Table 1). The expression of AQP4 in the brains of patients with MDD was shown to be downregulated by different groups [locus coeruleus (Bernard et al., 2011), hippocampus (Medina et al., 2016)]. However, in an oligonucleotide microarray analysis and qPCR study of postmortem brain tissues from patients with mood disorder, the expression of the AQP4 gene U34646 was upregulated in the prefrontal cortex (Iwamoto et al., 2004).

**TABLE 1:** Studies of astrocytes in postmortem tissues.

References	Pathological type	Brain regions of concern	Result
Torres-Platas et al., 2016	Depression	Mediodorsal thalamus and caudate nucleus	Downregulation of the GFAP mRNA
Gittins and Harrison, 2011	Mood disorder	Anterior cingulate cortex	Decreased GFAP protein levels
Cobb et al., 2016	MDD	Left hippocampus	Decreased number of GFAP-positive astrocytes
Altshuler et al., 2010	MDD	Amygdala	Decreased number of GFAP-positive astrocytes
Bernard et al., 2011	MDD	Locus coeruleus	Downregulated GFAP expression
Barley et al., 2009	MDD	Basal ganglia	Upregulated GFAP expression
Williams et al., 2013	Mood disorder	Cingulate cortex	Unchanged density of astrocytes
Feresten et al., 2013	BD	BA9	Increased GFAP expression
Webster et al., 2005	BD	Cingulate cortex	Decreased level of the GFAP mRNA
Johnston-Wilson et al., 2000	BD	BA10	Decreased number of GFAP-positive astrocytes
Toro et al., 2006	BD	BA11/47	Decreased number of GFAP-positive astrocytes
Fatemi et al., 2004	Mood disorder	Cerebellum	Decreased GFAP protein levels
Hercher et al., 2014	BD	Frontal cortex	Unchanged density of astrocytes
Pantazopoulos et al., 2010	BD	Amygdala and entorhinal cortex	Unchanged density of astrocytes

*GFAP, glial fibrillary acidic protein; MDD, major depressive disorder; BD, bipolar disorder; BA9, Brodmann Area 9; BA10, Brodmann Area 10; BA11/47, Brodmann Area 11/47.*

Similar to depression, astrocyte dysfunction is a very common characteristic of the occurrence and development of BD, but several experimental results show different trends. The density of astrocytes in the frontal cortex (Hercher et al., 2014), cingulate cortex (Williams et al., 2013), amygdala (Altshuler et al., 2010; Pantazopoulos et al., 2010), and entorhinal cortex (Pantazopoulos et al., 2010) did not change after death. However, some researchers have reached different conclusions: GFAP-positive astrocytes showed a decreasing trend in the BA10 (Johnston-Wilson et al., 2000) and BA11/47 (Toro et al., 2006) regions of the brain; and GFAP mRNA (Webster et al., 2005) or protein (Fatemi et al., 2004) expression decreased (see Table 1). Central nervous system-specific protein (S100β) is a calcium-binding protein that is mainly produced and secreted by astrocytes in the central nervous system. S100β is a commonly used astrocyte marker, and its level is increased in BA40 but decreased in BA9 (Dean et al., 2006). In a meta-analysis of serum S100β levels, serum levels of S100β were increased in patients with BD during the manic state (da Rosa et al., 2016).

Based on these experiments, abnormal astrocytes and AQP4 expression are very common characteristics of the pathology of mood disorders, which may be the main cause of glymphatic system dysfunction, but the contradictory experimental results require further integration and analysis.

### Headache

Headache, especially migraine, is closely related to mood disorders. The prevalence rate of migraine in clinical samples of patients with BD is 34.8% (Fornaro and Stubbs, 2015). At the same time, a high coprevalence of migraine and depression has been reported (Antonaci et al., 2011). Sleep interruption is a frequently reported cause of migraine (Wang et al., 2013), and sleep is often reported as an effective method to ameliorate migraine (Singh and Sahota, 2013). Calcitonin gene-related peptide (CGRP) is the core of the pathogenesis of migraine (Edvinsson, 2017). CGRP is released not only by the afferent nerve of the trigeminal nerve innervating the meninges, pia mater and internal cerebral arteries but also by the central projection of the spinal nucleus of the trigeminal nerve at the medulla oblongata level (Messlinger et al., 1993; Lennerz et al., 2008; Eftekhari et al., 2010). Although the exact mechanism of migraine caused by CGRP remains unclear, the fact that antibodies against CGRP or its receptor have been approved for clinical treatment (Yuan et al., 2017) indicate that CGRP plays an important role in the occurrence and development of migraine. CGRP released from the trigeminal afferent cannot does not the blood brain barrier, and it may be transferred to or cleared by the glymphatic system. After dural afferents are activated by depolarized KCl, the concentration of CGRP in CSF is 5 times higher than that in plasma (Dux et al., 2017). Since the clearance of metabolites, peptides and proteins from the brain by the glymphatic system mainly occurs during sleep, we speculate that sleep disorders cause glymphatic system disorders, resulting in the accumulation of CGRP in the brain and subsequently the symptoms of migraine.

Cortical spreading inhibition (CSD) is considered the neurophysiological basis of migraine aura (Bolay et al., 2002). Schain et al. (2017) initiated CSD by either pinprick or KCl crystals (prefrontal cortex), which led to rapid and almost complete closure of the space around the artery that gradually recovered within 30 min. In this mouse model, CSD caused temporary glymphatic flow disorders, and the direct relationship between the glymphatic system and migraine was proven. This finding may supplement the pathophysiological mechanism of migraine symptoms caused by glymphatic system dysfunction.

## Imaging Study

One of the main reasons for the doubt or contradictions in related research is the lack of mature imaging technology for glymphatic system. In recent years, a large number of studies have tried to use visualization technology to observe and describe the glymphatic system. The initial study used two-photon imaging observations, including fluorescent tracers and laser scanning microscopes, to discover the relationship between the glymphatic system and sleep (Xie et al., 2013) and found that alcoholism may lead to glymphatic system dysfunction (Liu Q. et al., 2020). Although optical imaging technology, such as two-photon microscopy, occupies a dominant position in the classical field, it is gradually being replaced by MRI because of its strong invasiveness.

MRI methods have been proven to be useful for the evaluation of glymphatic system in many experiments (Jiang, 2019; Taoka and Naganawa, 2020a,b). At present, the most commonly used MRI contrast agent is gadolinium-based contrast agent (GBCA). Iliff et al. (2013a) performed an intrathecal injection of the paramagnetic contrast agent GBCA and used dynamic contrast-enhanced MRI to display glymphatic function in the brains of living rats. This method characterizes the dynamic and spatial distribution of the glymphatic system in the whole brain. Because MRI has the least interference with the living body, similar methods have been used to study the glymphatic system in human subjects. A glymphatic system in humans similar to that found in the brains of rodents has been identified (Eide et al., 2018; Ringstad et al., 2018; Watts et al., 2019). Ringstad et al. (2017) used intrathecal GBCA as a CSF/ISF tracer for MRI and concluded that the glymphatic clearance rate was decreased in patients with iNPH. van de Haar et al. (2016) also used GBCA injection to confirm that the global blood-brain barrier leakage in patients with early AD was related to the decline of cognitive ability, reflecting the role of glymphatic system dysfunction in the pathological development of AD. Taoka et al. (2018) conducted animal experiments with intravenous injection of GBCA and found that the distribution of GBCA in brain tissue was affected by circadian rhythm and anesthesia. Currently, a variety of MRI methods have been developed to evaluate CSF movement, such as MRI tracer studies using the stable isotope ^17^O (Igarashi et al., 2014), multiple echo time (multi-TE) arterial spin labeling (ASL) MRI techniques (Ohene et al., 2019), and DTI-Alps methods to evaluate the movement of water molecules in the space around blood vessels by measuring the diffusion coefficient (Taoka et al., 2017). Abnormal glymphatic system function has been observed in the pathological processes of idiopathic normal pressure hydrocephalus (Ringstad et al., 2017; Eide and Ringstad, 2019), stroke (Gaberel et al., 2014), type 2 diabetes (Jiang et al., 2017; Yang et al., 2020), and juvenile myoclonic epilepsy (Lee et al., 2021). Different research groups have used MRI data for mathematical modeling to better understand the dynamics of glymphatic system flow (Kaur et al., 2020).

However, to date, no imaging technique has been established to directly and clearly depict the glymphatic system and neurofluid dynamics (especially in human experiments). Intrathecal injection of large doses of GBCA may cause severe gadolinium encephalopathy, potentially resulting in nausea, dyspnea, spasmodic pain of the lower extremities, and even death (Samardzic and Thamburaj, 2015; Reeves et al., 2017; Provenzano et al., 2019). Future neuroscience research is needed to develop increasingly precise measurement techniques for glymphatic system with less of an effect on normal physiological homeostasis and to improve relevant theories by obtaining quantitative data on various components of the glymphatic system as a method to obtain a deeper understanding of the relationship between the glymphatic system and various diseases.

## Conclusion and Prospects for the Future

Although glymphatic system dysfunction have been observed in individuals with a variety of pathological conditions, including central nervous system diseases and mood disorders, researchers have not clearly determined whether glymphatic system damage is the cause or result of these diseases (or both) (Mestre et al., 2017). The relationship between glymphatic dysfunction and diseases, as well as the specific underlying pathophysiological processes, are not understood. In recent years, the concept of “CNS interstitial fluidopathy” has been put forward by researchers, which refers to a variety of diseases characterized by glymphatic system dysfunction or other mechanisms related to interstitial fluid dynamics (Taoka and Naganawa, 2021). The in-depth study of the relationship between these diseases and glymphatic system will not only deepen our understanding of the glymphatic system and the pathogenic mechanism of related diseases, but also promote the development of clinical treatment and prevention strategies.

In addition, some doubts persist about the glymphatic system hypothesis. First, the model based on the Navier–Stokes equation and convection-diffusion equation related to fluid motion shows that the flow resistance of water molecules through the terminal AQP4 channel on astrocytes is too large to cause a large amount of fluid flow in the interstitial space, and thus researchers have questioned the regulatory role of AQP4 in CSF flow (Jin et al., 2016). Second, regarding the driving force of the glymphatic system, Asgari et al. (2016) used mathematical modeling to prove that the arterial pulse in the tissue is too small to be the driving force for large amounts of fluid flow, while the effects of other influencing factors, such as respiration and body position, must be further clarified. Third, other processes to remove waste in the brain might exist, such as transport across the blood brain barrier (Abbott et al., 2010), degradation mediated by enzymes expressed by astrocytes (Yin et al., 2006), and macrophage uptake (Hawkes and McLaurin, 2009), but the relationship between the glymphatic system and these pathways is still unknown. Therefore, future research should focus on elucidating the driving force of the glymphatic system and whether other possible driving factors exist, as well as their effects on the glymphatic system.

Sleep and endocrine factors play important roles in regulating the brain and peripheral organs. Sleep deprivation will increase the peripheral distribution of inflammatory markers in humans and rodents (Mullington et al., 2010; Hurtado-Alvarado et al., 2013; Irwin et al., 2016), while pathological signals from the brain promote systemic inflammation through the VEGF-C/VEGFR3 signaling pathway (Esposito et al., 2019). The characteristics of brain aging include disorders of mitochondrial energy metabolism, activation of inflammatory bodies, oxidative damage, autophagy defects, inflammation, and impaired waste disposal mechanisms (Franceschi et al., 2018; Mészáros et al., 2020). The normal physiological process of aging might also cause glymphatic system dysfunction through sleep disorders and endocrine abnormalities. The change in the normal sleep pattern is directly related to aging (Mander et al., 2017), and this change may inhibit the clearance function of the glymphatic system by reducing NREM sleep (Hablitz et al., 2019) and increasing AQP4 mislocalization (Kress et al., 2014). The normal aging process also leads to chronic changes in the phenotype of microglia similar to the proinflammatory response (Tay et al., 2017), and activated microglia are part of the process inducing the formation of A1-like reactive astrocytes (Clarke et al., 2018), coupled with the cellular senescence characteristics of astrocytes themselves, such as increased levels of GFAP (Cotrina and Nedergaard, 2002; Finch, 2003) and vimentin filaments (Clarke et al., 2018); increased expression of cytokines such as tumor necrosis factor α (TNF- α), IL-1b and IL-6 (Campuzano et al., 2009); and increased accumulation of proteotoxic aggregates (Clarke et al., 2018). Lipids may also be secreted by astrocytes in an autocrine fashion, into PVSs, potentially to signal to other astrocytes, ultimately effecting ion fluxes and downstream phosphorylation pathways that themselves contribute to modulating the release or uptake of many other signaling agents (Rangroo Thrane et al., 2013). Therefore, abnormal astrocytes may affect the neural networks macroscopically. These changes may affect the normal operation of the macroscopic waste removal system in the brain through the endocrine pathway. Since sleep disorders and endocrine disorders caused by aging involve both brain and peripheral regulation and glymphatic system function, we infer that the glymphatic system plays a certain role in regulating the brain and peripheral organs. However, the role of the glymphatic system requires further study in the future.

## Author Contributions

BL made the conception and design of the work, and revised the manuscript draft. DZ and XL reviewed the literature and wrote the manuscript draft. All authors contributed to the article and approved the submitted version.

## Conflict of Interest

The authors declare that the research was conducted in the absence of any commercial or financial relationships that could be construed as a potential conflict of interest.

## Publisher’s Note

All claims expressed in this article are solely those of the authors and do not necessarily represent those of their affiliated organizations, or those of the publisher, the editors and the reviewers. Any product that may be evaluated in this article, or claim that may be made by its manufacturer, is not guaranteed or endorsed by the publisher.
